# Negative repercussions of caregiving burden: Poor psychological well-being and depression

**DOI:** 10.12669/pjms.346.15915

**Published:** 2018

**Authors:** Neelam Ehsan, Nigar Johar, Tamkeen Saleem, Muhammad Alamgir Khan, Samia Ghauri

**Affiliations:** 1 *Dr. Neelam Ehsan, Assistant Professor, Department of Psychology International Islamic University, Islamabad, Pakistan*; 2 *Maj. Gen. Dr. Nigar Johar, Vice Principal, Army Medical College, Rawalpindi, Pakistan*; 3 *Dr. Tamkeen Saleem, Assistant Professor, Department of Psychology International Islamic University, Islamabad, Pakistan*; 4 *Muhammad Alamgir Khan, Professor of Physiology, Army Medical College, Rawalpindi, Pakistan*; 5 *Ms. Samia Ghauri, Trainee. Masters Psychology, Department of Psychology International Islamic University, Islamabad, Pakistan*

**Keywords:** Caregiver, Depression, Psychological Well-being

## Abstract

**Objectives:**

To compare depression and psychological well-being between caregivers of schizophrenic patients and non-caregivers and to study the burden of caregiving as a relative risk for depression and psychological well-being.

**Methods:**

This cross sectional comparative study was conducted at International Islamic university Islamabad from January to September 2017. Fifty informal caregivers of schizophrenic patients from 19 to 55 years of age were included in the study. The control group consisted of age and socio-economic status matched healthy volunteers who did not have any psychological or medical patient at home needing care and assistance. For measurement of study variables i.e., burden of caregiving, depression and psychological well-being, instruments used were Zarit Burden Interview (ZBI), The Beck Depression Inventory (BDI) and Warwick-Edinburgh Mental Well-Being Scale (WEMWBS) respectively.

**Results:**

Results were analyzed using MANOVA followed by One-Way ANOVA. Findings indicated that informal caregivers of schizophrenia have greater depression and poor psychological well-being in comparison to the non-caregiver controls. Association of caregiving burden with psychological well-being and depression was calculated using Chi Square test and relative risk.

**Conclusion:**

Caregiving adversely affects informal caregivers’ mental health and wellbeing. Informal caregiving is a burden for the caregivers; health status of family members involved in caregiving should be routinely assessed to enhance their health-related quality of life.

## INTRODUCTION

According to the World Health Organization^1^ approximately 24 million people between 15-20 years of age, experience schizophrenia, and overall, 450 million people worldwide are fighting with mental illnesses. Most of such people are dependent on their families to take care of them and help them out in their daily activities of life. Caregivers are persons within the family of patient, who are responsible for looking after the patient’s health, daily activities and well-being. Considering the nature of mental illnesses, they remain in constant interaction with patients for a long time which puts them under substantial psychological burden. The World Health Organization^2^ states that “the fulfillment of responsibility and physical, financial and emotional needs of an ill person carried out by a person that is not a part of any health care system is regarded as the caregiver’s burden who can be a family member or a close friend.” Care giving burden is defined as a load of difficulties for the people who take care of a psychiatric patient.^3^ There are two types of care giving burden. Objective burden is the negative effects or patient’s negative symptoms and disturbance in day-to-day tasks of the caregiver. On the contrary, subjective burden is the emotional stress of the caregiver like fear, anger, sadness, etc. The burden of persons with mental illness is estimated as 14% around the globe with highest figures in developing countries.^4^

Caregiving to mentally ill member of the family impacts the family members in general and the primary caregivers in particular. Caregiving to such patients can affect caregivers physically and psychologically.^5^ Caregivers generally experience; feelings of stress, bitterness and enervation that affect their wellbeing. In a marital relationship where emotional needs are satisfied but if one of the spouse is mentally ill then appropriate affection and attention is not provided. Moreover, presence of a mentally ill family member creates disturbances in interacting with others.^6^ It was revealed that the major supporting pillar for the mentally ill people in the community is their family who continue taking care of their loved ones.^7^ Most of the time caregivers stay busy in taking care for an ill family member due to which their emotional and physical needs are neglected and sacrificed that can significantly impair their psychological wellbeing.^3^ In comparison to the common population the levels of depression is high in the caregivers as it is an emotionally, psychologically and physically exhausting experience. The World Health Organization anticipated that in 2020 the most leading and commonly seen health disorder would be depression.^8^ Another study recommended 60% caregivers to reschedule their daily routine to avoid conflicts such as; being unpaid for absences and working for less than normal hours of duty.^9^ Literature highlights that approximately 19% (or one in five) people are caregivers of more than one mentally ill patient in family. Four of ten patients lived with their caregivers in the same house. People who cared for their son or daughter were 76% whereas, 7% were caregivers of their brothers or sisters and 10% were involved in caregiving of their wife or husband.^10^

Caregivers of schizophrenics and mood disorders patients are usually their own parents, mostly mothers who too are often aged. Having a schizophrenic spouse adds to the burden of caregiver, along with the household responsibilities. All the responsibilities are to be fulfilled mostly without any external source of help. Apart from the spouse; emotional, financial, social and bodily burden due to a schizophrenic patient was also reported in some close relatives.^11^ Psychiatric symptoms leading to burden in care giving were of lower levels only when the caregiver and mentally ill family member had love and intimacy in their relationship.^12^ Treatment of mental disorders (e.g. schizophrenia or bipolar disorder) requires intensive intervention and care. Caregivers are involved in the energetic and dynamic procedures of long term care giving which in-turn makes the caregiver feel burdened. Current study was planned to find out the effects of care giving burden on psychological wellbeing and depression in caregivers of the schizophrenic patients.

## METHODS

The present cohort retrospective study was conducted at International Islamic University Islamabad from January 2017 to September 2017. The Ethical Approval was granted by the Ethical Review Committee of the institution. Sample size was calculated using WHO sample size calculator. A sample of 100 individuals, 50 informal caregivers of schizophrenic patients and 50 non-care giver controls were determined for the present study by keeping alpha value as 0.05 and power of test as 0.8, standard deviation as 9.0 and difference of the means as 5.0. The control group consisted of age and socio-economic status matched healthy volunteers who did not have any psychological or medical patient at home needing care and assistance.

The data for non-care giving controls was collected from International Islamic University Islamabad and Shaheed Benazir Bhutto University. The sample for caregivers of schizophrenic patients was collected from psychiatry wards of Lady Reading Hospital and Khyber Teaching Hospital Peshawar. An exclusion criterion for the present study was prior history of any psychiatric illness for the caregivers. Data was collected after getting the approval from the institutional authorities. The participants were approached and explained about the purpose of the research. They were assured that the results of the study will be only utilized for the sake of research and their responses and identity will be kept confidential. Their written informed consent was taken for participation. The participants were provided with the study questionnaires along with the instructions and their queries were entertained. Each participant was attended individually and was thanked for his/her participation in the study.

The data collection instruments used comprised of a data sheet measuring demographic variables like age, gender and family type. Zarit Care giving Burden Scale was used for measuring care giving burden.^13^ The Scale was translated in Urdu language as many of our participants were unable to comprehend it in English. The ‘Urdu version’ of the scale was reviewed by two experts to ensure consistency with the original scale. The Beck Depression Inventory (BDI) was used for assessing depression levels among the sample.^14^ It consists of 21items having four possible responses for each item according to the intensity of the symptoms. The alpha coefficient of the scale is 0.86 whereas the test-retest reliability is 0.93. Total BDI score were converted into a dichotomous variable keeping a cut off value at 16 (no depression if the score was equal to or below 16 and depression if the score was over 16). Psychological wellbeing among the sample was assessed by Warwick-Edinburgh Mental Well-Being Scale (WEMWBS).^15^ It is a 14 item scale with response options ranging from one (none of the time) to five (all of the time). The minimum score on the scale is 14 and maximum is 70. The scale is validated for use with those aged 16 years and above. It has Cronbach alpha reliability of 0.89 and test-retest reliability of 0.83. Total score on Warwick-Edinburgh Mental Well-Being Scale was also converted into a dichotomous variable considering a cut off value of 35 (bad psychological wellbeing at score 35 or below and good if the score is more than 35). The data were analyzed using SPSS version 25. Mean and standard deviations were calculated for numerical variables whereas frequency and percentage for categorical variables. Multivariate analysis of variance (MANOVA) followed by One-Way ANOVA was performed to determine the effect of care giving burden on psychological wellbeing and depression. Association of care giving burden with psychological wellbeing and depression was calculated using Chi Square test and Relative Risk. Alpha was kept at 0.05.

## RESULTS

There were 20 (42.6%) males and 30 (56.6%) females in care giving group whereas 27 (57.4%) males and 23 (43.4%) females in non-care giving group. Frequency difference of the two genders between the two groups was non-significant (*p*-value=0.16). Mean age of participants in care giving groups was 31.76±9.13 years with range of 19-55, whereas that for non-care giving group was 35.72±9.69 with range of 23-55 years.

Multivariate analysis of variance showed that effect of care giving was significant on psychological wellbeing and depression when considered jointly (Wilk Lambda = 0.53, *F* (2, 97) = 43.05, *p* = <0.001, partial eta squared = 0.47. Comparison of psychological wellbeing and depression separately between the two levels of independent factor (care giving) are shown in Table-I.

**Table-I:** Comparison of psychological wellbeing and depression between care giving and non-care giving groups n.

Variables	Groups	Mean±SD	p-value	Partial eta squared
Psychological wellbeing	Care giving (n=50)	21.16±10.66	<0.001^*^	0.36
	Non-care giving (n=50)	37.72±11.67		
Depression	Care giving (n=50)	26.08±9.49	<0.001^*^	0.45
	Non-care giving (n=50)	9.28±9.37		

Frequency comparison of the two categories of psychological wellbeing between care givers and non-care givers along with the p-value is shown in Table-II. The table also shows relative risk along with 95% confidence interval.

**Table-II:** Association of Care giving burden with Psychological Well-being

Groups	Psychological well-being		p-value	Relative Risk	

	Poor	Good		Value	95% confidence interval
Care givers	45 (90%)	5 (10%)	<0.001^*^	2.14	1.53 – 3.00
Non-care givers	21 (42%)	29 (58%)			

Frequency comparison of individuals with and without depression between care givers and non-care givers is shown in Table-III. Table also shows p-value and relative risk along with 95% confidence interval.

**Table-III:** Association of care giving burden with depression.

	Depression		p-value	Relative Risk	
	Yes	No		Value	95% CI
Care givers	40 (80%)	10 (20%)	<0.001^*^	6.67	3.10 – 14.30
Non-care givers	6 (12%)	44 (88%)			

## DISCUSSION

The study aimed to find out association of care giving burden with psychological wellbeing and depression in caregivers of schizophrenic patients. Results of the study revealed that the risk of bad psychological wellbeing was two times more whereas that of depression was about seven times more in caregivers of schizophrenic patients as compared to healthy controls. This enormous magnitude of association is alarming especially considering the increasing prevalence of psychological disorders. At times, the ratio of caregivers to care recipient patient is more than one leading to increase in the number of family members who are in psychological bad health and depression. This, ultimately, puts the whole family at risk of developing psychological illnesses.

Our study findings are supported by previous studies. Pinquart reported that care giving burden was significantly associated with depression, subjective wellbeing and stress.^16^ Another study documented that the caregivers were less satisfied and had more negative affect as compared to the non-caregivers regardless of their marital status and age.^17^

Living with and providing care to mentally ill patients is associated with adverse effects on wellbeing of care givers.^18^ The caregivers have to bear with the behavioral disturbances like violent and abusive behaviors of ill member. As a result, in many cases caregivers have to curtail on their social activities including their jobs. Thus, care giving leads to being stressed out and anxious because the nature of illness is chronic and demanding.^19^ Higher risk of depression and poor psychological wellbeing in caregivers of mentally ill patients is a serious societal issue and needs to be addressed in a befitting manner. Care giving burden is a multi-faceted stressor that creates high level of burden which contributes to depressive symptoms.^20^ According to Australian Bureau of Statistics, well-being of 30% of caregivers was affected due to care giving and they also reported worry and depression more often in their lives.^21^ A study reported that nearly one quarter of caregivers is at risk of developing depression while in their care giving role. It was further added that this group had never been diagnosed with depression prior to the assumption of care giving role.^22^ Moreover, it was also observed that depression and perceived burden among caregivers significantly increased as the care recipient’s functional status declined.^23^

Providing care to psychologically ill patients is challenging and this makes the caregivers a high-risk group for developing depression and bad mental health. This ‘high-risk’ group of caregivers needs to be put under medical surveillance for timely intervention of any adverse outcomes. The situation becomes serious when the caregivers drift silently into depression and poor mental health without the actual cause being noticed. This ultimately consumes the whole family adversely affecting all the aspects of human lives.

### Limitations

The sample size of study was small due to the chronicity and complexity of schizophrenia. Schizophrenia requires a thorough assessment for diagnosis on behalf of the clinician. Our study included caregivers of only diagnosed patients of schizophrenia. However, it is suggested for the future studies to recruit a comparatively larger sample in order to increase generalizability.

## CONCLUSION

Caregivers of schizophrenic patients are at high risk of developing poor psychological wellbeing and depression. This subset of ‘potential patients’, needs special care and support from other family members, society and health care providers to prevent them develop psychological illnesses. This would be an essential step towards reducing the burden of psychological illnesses from the society.

### Author’s Contribution

**NJ:** Designed the study, Approval of the version to be published.

**TK:** Contributions to idea and designing, Collection, Analysis and Interpretation of data, Preparation of manuscript.

**NE:** Designing, Collection, Analysis and Interpretation of data, Revising the article, Approval of the manuscript.

**MAK:** Statistical data analysis. Writing of results, discussion and methodology.

**SG:** Data collection.

All authors have approved the final manuscript for publication.
